# Effect of Dietary Content of Menhaden Oil with or without Salsalate on Neuropathic Endpoints in High-Fat-Fed/Low-Dose Streptozotocin-Treated Sprague Dawley Rats

**DOI:** 10.1155/2018/2967127

**Published:** 2018-07-02

**Authors:** Eric P. Davidson, Lawrence J. Coppey, Hanna Shevalye, Alexander Obrosov, Mark A. Yorek

**Affiliations:** ^1^Department of Internal Medicine, University of Iowa, Iowa City, IA 52242, USA; ^2^Department of Veterans Affairs Iowa City Health Care System, Iowa City, IA 52246, USA; ^3^Fraternal Order of Eagles Diabetes Research Center, University of Iowa, Iowa City, IA 52242, USA

## Abstract

In this study, we wanted to extend our investigation of the efficacy of fish oil with or without salsalate on vascular and neural complications using a type 2 diabetic rat model. Four weeks after the onset of hyperglycemia, diabetic rats were treated via the diet with 3 different amounts of menhaden oil with or without salsalate for 12 weeks. Afterwards, vascular reactivity of epineurial arterioles and neuropathy-related endpoints were examined. The addition of salsalate to high-fat diets enriched with 10% or 25% kcal of menhaden oil protected vascular reactivity to acetylcholine and calcium gene-related peptide, motor and sensory nerve conduction velocity, thermal nociception, intraepidermal nerve fiber density, and cornea sensitivity to a greater extent than 10% or 25% menhaden oil alone. Vascular and neural function was maximally protected with diet containing 45% kcal as menhaden oil, and adding salsalate did not provide any additional benefit. Salsalate alone in the high-fat diet of diabetic rats provided minimal protection/improvement of vascular and neural dysfunction. These studies imply that dietary salsalate in combination with lower amounts of menhaden oil can provide greater benefit toward diabetes-induced vascular and neural impairment than menhaden oil alone.

## 1. Introduction

Peripheral neuropathy is one of the most common complications of diabetes and has been reported to affect about 50% of the diabetic population, and up to 30% of the population reported to be prediabetic with insulin resistance [1, 2]. After many years of research, there is no treatment for diabetic peripheral neuropathy other than good glycemic control but for patients with the most common form of diabetes, referred to as late onset or type 2 diabetes, good glycemic control provides minimal benefit [1]. Therefore, there is a critical need for a treatment that will at the minimum slow progression.

Previously, we have demonstrated that increasing the dietary content of fish oil alone or in combination is an effective treatment for vascular and neural complications in diabetic rodents [3, 4]. We have also shown that cotreatment of type 1 diabetic mice with menhaden (fish) oil and salsalate is more effective toward neuropathy than menhaden oil alone and that circulating resolvin D1 levels are increased when salsalate is combined with menhaden oil [5]. Lastly, we have demonstrated that treating either type 1 or type 2 diabetic mice with daily injections of resolvin E1 or D1, metabolites of eicosapentaenoic acid (EPA) and docosahexaenoic acid (DHA), respectively, most abundant omega-3 polyunsaturated fatty acids found in fish oil, improves peripheral neuropathy [6, 7].

Increasing consumption of omega-3 (n-3) polyunsaturated fatty acids, commonly found in marine mammals, through the diet or by supplements has been shown to improve cardiovascular disease [8, 9]. Decreasing the n-6 to n-3 ratio of polyunsaturated fatty acids in circulation has been linked to reducing inflammatory stress [10, 11]. Furthermore, several metabolites of EPA and DHA including neuroprotectin D1 and E and D series resolvins have been shown to have anti-inflammatory and neuroprotective properties [6, 12, 13]. There have been very few side effects reported following increased natural consumption of n-3 polyunsaturated fatty acids. Individuals increasing fish oil consumption via capsules have reported a fishy taste or breath, and no adverse events were reported in a large multicenter clinical trial [14]. Therefore, increasing intake of n-3 polyunsaturated fatty acids is widely considered to be safe and potentially beneficial toward different metabolism-related disorders. Moreover, it has been reported that a higher n-3 polyunsaturated fatty acid index is associated with statistically significant, clinically relevant lower systolic and diastolic blood pressure levels in normotensive young and healthy individuals [15]. From studies in animal models, it would seem that increasing the formation of metabolites of n-3 polyunsaturated fatty acids would also be an advantageous approach to improving health benefits of increased intake of EPA and DHA. To that end, we have investigated the potential for salsalate to increase production of resolvins and its effect on vascular reactivity and neural endpoints related to diabetic peripheral neuropathy.

Salicylsalicylic acid (salsalate), a nonacetylated salicylate, is a nonsteroidal anti-inflammatory agent that inhibits the synthesis of prostaglandins by inactivating cyclooxygenase enzymes [16]. Salsalate is insoluble in gastric juice and moves to the small intestine where it is hydrolyzed into two salicylic acid molecules. In clinical trials, the main side effects of salsalate were tinnitus, headache, dizziness, and gastrointestinal discomfort, which occurred at higher doses and subsided following withdrawal of treatment [17, 18]. Salsalate produces less gastrointestinal bleedings when compared to acetylsalicylic acid due to less mucosal prostaglandin inhibition [19].

In this study, we have used a rat model of type 2 diabetes and examined the singular and combined effect of salsalate with 3 different concentrations of menhaden oil on vascular reactivity by epineurial arterioles, blood vessels that provide circulation to the sciatic nerve and multiple endpoints associated with peripheral nerve function.

## 2. Materials and Methods

Chemicals used in this study came from Sigma-Aldrich chemical company (St. Louis, MO, USA), unless otherwise stated.

### 2.1. Animals

Sprague Dawley (Envigo, Indianapolis, IN) male rats 10–11 weeks of age were housed in an Association for Assessment and Accreditation of Laboratory Animal Care accredited animal care facility, and food (Envigo, number 7001, 4.25% kcal as fat, 3.0 kcal/g, Madison, WI) and water were provided ad libitum. These studies were approved by the University of Iowa Animal Care and Use Committee (number 5071450). All institutional and NIH guidelines for use of animals were followed. At 12 weeks of age, rats were divided into 8 groups. Rats in seven of these groups were designated to become diabetic and were placed on a high-fat diet (D12451 [45% kcal as fat, 4.7 kcal/g], Research Diets, New Brunswick, NJ) with lard as the primary source of the fat. The remaining set of rats were designated to be the control group and remained on the base diet throughout the study. Eight weeks later, the high-fat-fed rats were treated with streptozotocin (30 mg/kg in 0.1 M citric acid buffer, pH 4.5, ip, EMD/Millipore, Billerica, MA) to induce hyperglycemia. Control rats were treated with vehicle. Blood glucose was evaluated 96 h later using glucose-oxidase reagent strips (Aviva Accu-Chek, Roche, Mannheim, Germany), and rats having a blood glucose level of 250 mg/dl (13.8 mM) or greater were considered to be diabetic. Four weeks after the confirmation of hyperglycemia, treatments were initiated. Rats in the untreated group continued to receive the 45% kcal high-fat diet. One treatment group received the 45% kcal high-fat diet supplemented with 2 g/kg salsalate. Three treatment groups received a high-fat diet with 22%, 56%, or 100% of the kcal derived from lard replaced with menhaden oil. These groups were referred to as 10%, 25%, and 45% menhaden oil-treated rats, respectively. The three remaining treatment groups received the three different menhaden oil-enriched diets supplemented with 2 g/kg salsalate. These modified high-fat diets were prepared by Research Diets. The final fat content of all the high-fat diets was maintained at 45% kcal through a mixture of lard and/or menhaden oil as the source of the fat. The treatment period was 12 weeks.

### 2.2. Nerve Function Endpoints

Thermal nociceptive response was assessed in rats one week prior to euthanasia using the Hargreaves method with an IITC Life Science Inc. device (Woodland Hills, CA, model 390G) as previously described [20]. Prior to measurement, rats were allowed 15 min to assimilate in the device. Six recordings were made using both hindpaws with a 5-minute delay between recordings, the initial recording was discarded, and the remaining five were averaged, presented in seconds, and served as the thermal nociceptive response latency. An automatic termination of the operation was set for 25 sec to avoid tissue injury to the rat due to severe decrease of sensation.

Corneal sensation was determined by a Cochet-Bonnet filament esthesiometer (Luneau Ophtalmologie, France) [21]. Rats were gently restrained by hand and the 6 cm filament advanced to touch the eye. If the rat blinked, the length of the filament was recorded. If the rat did not blink, the filament was shortened by 0.5 cm and the procedure was repeated. This process was continued until the rat blinked. Each eye was evaluated. The data were reported in cm.

On the day of terminal studies, rats were anesthetized with Nembutal (50 mg/kg, ip, Diamondback Drugs, Scottsdale, AZ) and motor and sensory nerve conduction velocities were assessed in the sciatic-posterior tibial conducting system and digital nerve, respectively, as in previous experiments [20]. Motor nerve conduction velocity was calculated by using the stimulus artifact of the evoked potential, subtracting the latency measurement (in milliseconds) from the sciatic notch from the latency measurement of the Achilles tendon, and dividing the difference by the distance between the two stimulating electrodes (measured in millimeters). Sensory nerve conduction velocity equaled the distance between stimulating and recording electrodes over the latency to initial peak negative deflection. Both motor and sensory nerve conduction velocity was reported in meters per second.

Subepithelial corneal nerves were imaged using the Rostock cornea module of the Heidelberg Retina Tomograph confocal microscope (Heidelberg Engineering, Heidelberg, Germany) [21]. The investigator acquiring these images was masked with respect to identity of the animal condition. Anesthetized rats were secured to a stereotaxic rat head holder mounted on a platform that allows for three-dimensional adjustments. GenTeal eye lubricant gel (Alcon; Fort Worth, TX) was applied to the lens, and the lens was advanced until the lubricant came into contact with the rat cornea epithelium. A minimum of six images was acquired for each animal by adjusting the platform to focus on a different region of the cornea. The data is presented as corneal nerve fiber length defined as the total length of all nerve fibers and branches (in millimeters) present in the acquired images standardized for area of the image (in square millimeters). The corneal fiber length for each animal was the mean value obtained from the acquired images and expressed as mm/mm^2^.

Immunoreactive nerve fiber profiles innervating the skin from the hindpaw were visualized using standard confocal microscopy combined with immunohistochemistry [20]. The primary antibody used was anti-tubulin B3 (BioLegend, San Diego, CA), and the secondary antibody was Alexa Fluor 546 goat anti-mouse (Life Technologies, Carlsbad, CA). Profiles were imaged and counted using a Zeiss LSM710 confocal microscope (Oberkochen, Germany) with a 40x objective by two individual investigators that were masked to the sample identity and normalized to length. Data are presented as profiles/mm.

Videomicroscopy was used to investigate in vitro vasodilatory responsiveness of epineurial arterioles, blood vessels that provide circulation to the region of the sciatic nerve [20]. Following isolation, suspension of the vessels in organ chambers, and verification of reactivity, cumulative concentration-response relationships were evaluated for acetylcholine (10^−8^–10^−4^ M) and calcitonin gene-related peptide (10^−11^–10^−8^ M). At the end of each dose response curve, papaverine (10^−5^ M) was added to determine maximal vasodilation.

### 2.3. Serum Determinations

Rat blood was collected from the heart's right ventricle, and serum was analyzed for triglycerides, free fatty acids, cholesterol, and resolvin D1 using commercial kits as reported in previous experiments [20].

### 2.4. Data Analysis

Results are presented as mean ± SEM. Comparisons between the treatment groups and control and nontreated diabetic rats were conducted using one-way ANOVA and Bonferroni posttest comparison (Prism software 7; GraphPad, San Diego, CA). Concentration response curves for acetylcholine and calcitonin gene-related peptide were compared using a two-way repeated-measures analysis of variance with autoregressive covariance structure using PROC MIXED program of SAS [20]. Log EC_50_ were generated using Prism software. A *P* value less than 0.05 was considered significant.

## 3. Results

**Figure 1:**
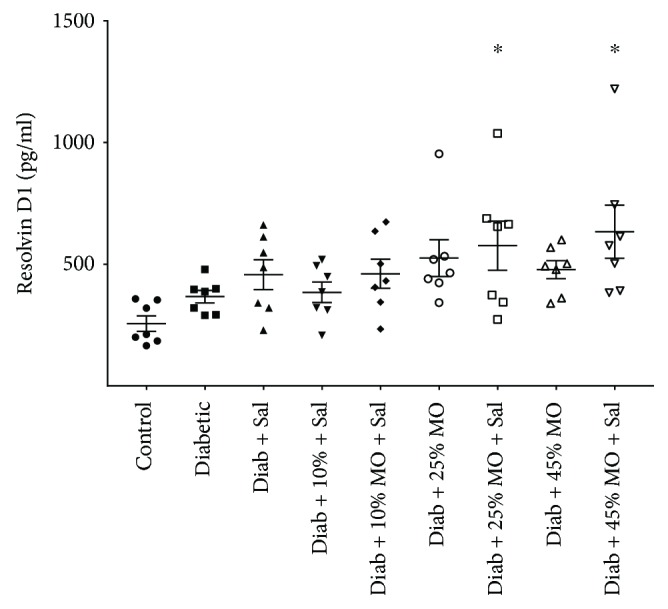
Scatter plot of serum resolvin D1 levels. Effect of menhaden oil concentration with or without salsalate on serum levels of resolvin D1 in type 2 diabetic rats are presented. Serum levels are presented as mean ± SEM and are in pg/ml serum. Each symbol per group represents a different animal, *n* = 7 per condition. ^∗^ *P* < 0.05 compared to control rats.

The weight of all rats at the start of the study was statistically the same (Table 1). All rats gained weight over the study period. There was a trend for the diabetic rats to gain less weight than the control rats, but the difference was not significant between any groups. There was no statistical difference in the amount of chow consumed by the rats in any of the groups. All diabetic rats were hyperglycemic at the end of the study, and no treatments changed the nonfasting blood glucose levels. Serum triglyceride levels were significantly increased in the untreated diabetic rats and diabetic rats treated with menhaden oil compared to control rats. The serum triglyceride levels in diabetic rats treated with 45% menhaden oil were significantly less than the serum triglyceride levels in untreated diabetic rats. Treating diabetic rats with salsalate with or without menhaden oil caused serum triglyceride levels to be lower at the end of the study compared to untreated diabetic rats. Serum triglyceride levels in diabetic rats treated with salsalate were higher than those observed in control rats, but the comparison was not statistically different. Serum-free fatty acid and cholesterol levels were significantly increased in untreated diabetic rats and diabetic rats treated with or without menhaden oil and/or salsalate compared to control rats with the exception of diabetic rats treated with 45% menhaden oil and salsalate. Serum resolvin D1 levels were measured in all groups of rats at the end of the study period. Data in Figure 1 demonstrate that resolvin D1 levels were significantly increased in diabetic rats treated with 25% and 45% menhaden oil with salsalate compared to control rats.

At the end of the study period, several endpoints related to peripheral nerve activity and density were examined (Table 2). Motor and sensory nerve conduction velocity was significantly decreased in untreated diabetic rats and diabetic rats treated with salsalate alone or with 10% or 25% menhaden oil compared to control rats. Treating diabetic rats with 10% menhaden oil and salsalate significantly improved motor nerve conduction velocity but not sensory nerve conduction velocity compared to untreated diabetic rats. Treating diabetic rats with 25% menhaden oil with salsalate significantly improved both motor and sensory nerve conduction velocity compared to untreated diabetic rats. Treating diabetic rats with 45% menhaden oil with or without salsalate significantly improved both motor and sensory nerve conduction velocity.

Thermal nociception was significantly impaired in untreated diabetic rats, and the difference was not statistically improved in diabetic rats treated with salsalate alone compared to control rats (Table 2). Treating diabetic rats with 10% menhaden oil moderately improved thermal nociception compared to control and untreated diabetic rats. However, treating diabetic rats with 10% menhaden oil with salsalate or with 25% or 45% menhaden oil with or without salsalate significantly improved thermal nociception compared to untreated diabetic rats. Intraepidermal nerve fiber density was significantly decreased in untreated diabetic rats, diabetic rats treated with salsalate alone, diabetic rats treated with 10% menhaden oil with or without salsalate, or diabetic rats treated with 25% menhaden oil. Treating diabetic rats with 25% menhaden oil with salsalate or with 45% menhaden oil with or without salsalate significantly improved intraepidermal nerve fiber density compared to untreated diabetic rats.

Determination of changes in corneal nerve sensitivity and subepithelial corneal nerve density is being promoted as an early marker of peripheral nerve damage in human subjects with diabetes [22, 23]. We have also been examining diabetes-induced changes in corneal nerve sensitivity and subepithelial corneal nerve density in diabetic rodents and found changes to correlate with other markers of peripheral neuropathy [24, 25]. In this study, we found that corneal sensitivity was significantly decreased in untreated diabetic rats, diabetic rats treated with salsalate alone, diabetic rats treated with 10% menhaden oil with or without salsalate, and diabetic rats treated with 25% menhaden oil compared to control rats (Table 2). In contrast, treating diabetic rats with 25% menhaden oil with salsalate or 45% menhaden oil with or without salsalate significantly improved corneal nerve sensitivity compared to untreated diabetic rats. Subepithelial corneal nerve fiber length was significantly decreased in untreated diabetic rats, diabetic rats treated with salsalate alone, and diabetic rats treated with 10% menhaden oil with or without salsalate compared to control rats. In contrast, subepithelial corneal nerve fiber length was protected significantly when diabetic rats were treated with 25% or 45% menhaden oil with or without salsalate compared to untreated diabetic rats.

**Figure 2:**
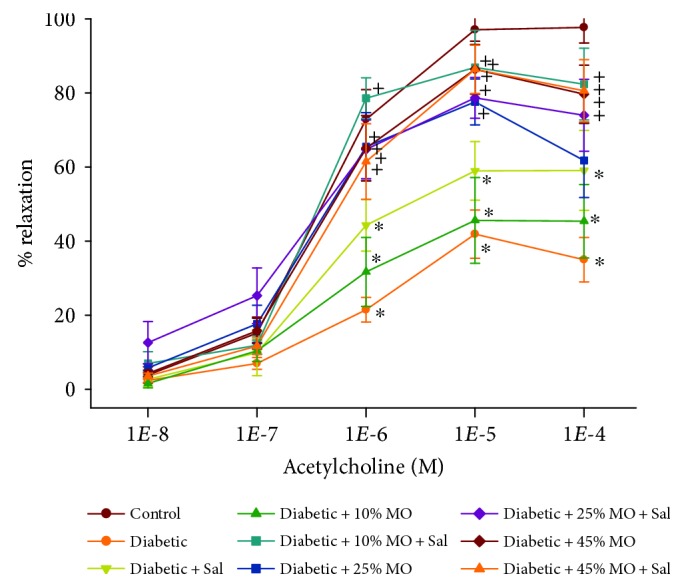
Effect of menhaden oil concentration with or without salsalate on vascular relaxation by acetylcholine in epineurial arterioles of the sciatic nerve in Sprague Dawley type 2 diabetic rats. Pressurized arterioles (40 mmHg and ranging from 60 to 100 *μ*m luminal diameters) were constricted with phenylephrine (30–50%), and incremental doses of acetylcholine were added to the bathing solution while recording steady-state vessel diameter. The number of rats in each group was the same as shown in Table 1. Data are presented as the mean of % relaxation ± SEM. ^∗^ *P* < 0.05 compared to control rats; ^+^*P* < 0.05 compared to diabetic rats.

**Figure 3:**
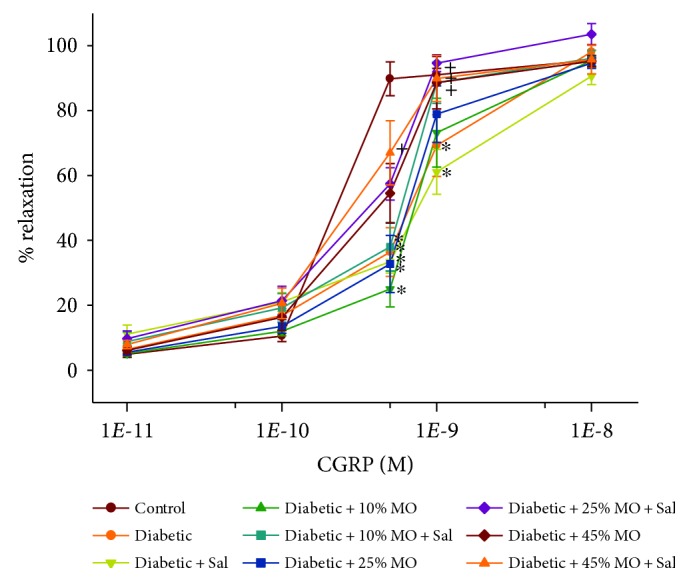
Effect of menhaden oil concentration with or without salsalate on vascular relaxation by calcitonin gene-related peptide in epineurial arterioles of the sciatic nerve in Sprague Dawley type 2 diabetic rats. Pressurized arterioles (40 mmHg and ranging from 60 to 100 *μ*m luminal diameters) were constricted with phenylephrine (30–50%), and incremental doses of calcitonin gene-related peptide were added to the bathing solution while recording steady-state vessel diameter. The number of rats in each group was the same as shown in Table 1. Data are presented as the mean of % relaxation ± SEM. ^∗^*P* < 0.05 compared to control rats; ^+^*P* < 0.05 compared to diabetic rats.

We have previously demonstrated that decreased vascular reactivity by epineurial arterioles, resistance size vessels that provide circulation to the sciatic nerve, to acetylcholine precedes decrease in motor nerve conduction velocity [26]. We have also shown that epineurial arterioles are innervated by sensory nerves expressing calcitonin gene-related peptide and that the calcitonin gene-related peptide is a very potent vasodilator of these vessels and this vasodilation is impaired by diabetes [27]. In this study, like in previous studies, we found that diabetes causes a decrease in vasodilation to acetylcholine (Figure 2 and Table 3). Treating diabetic rats with salsalate alone or with 10% menhaden oil did not significantly improve vascular relaxation to acetylcholine compared to untreated diabetic rats. However, treating diabetic rats with 10% menhaden oil with salsalate or 25% or 45% menhaden oil with or without salsalate significantly improved vascular relaxation. Vascular relaxation of epineurial arterioles to calcitonin gene-related peptide at 5 × 10^−10^ and 10^−9^ M doses was significantly decreased in untreated diabetic rats and was not improved when diabetic rats were treated with salsalate alone or with 10% menhaden oil (Figure 3 and Table 3). In contrast, treating diabetic rats with 10% menhaden oil with salsalate tended to improve vascular relaxation to the calcitonin gene-related peptide, and treating diabetic rats with 25% or 45% menhaden oil with or without salsalate significantly improved vascular relaxation to calcitonin gene-related peptide compared to untreated diabetic rats.

## 4. Discussion

These studies were performed in a high-fat diet fed rats treated 8 weeks later with a low dose of streptozotocin. This diabetic rat models late-stage type 2 diabetes. It has been reported to simulate the human syndrome and to be suitable for testing the effect of antidiabetic compounds [28, 29]. Furthermore, my laboratory previously has characterized the progression of peripheral neuropathy in this model including decrease in corneal nerve sensitivity and density [20, 21, 24]. Using this model, we have been seeking potential new treatments for diabetic vascular and neural complications that would be safe for human use and have minimal side effects. In the present experiments, we examined the therapeutic potential of salsalate with and without menhaden oil on diabetes-induced vascular and neural deficits. Both of these compounds have been shown to be safe for human use.

In humans with type 2 diabetes, salsalate, 3.5 g/day, improved glycemia and decreased inflammatory markers [17]. In obese, insulin-resistant, nondiabetic subjects, salsalate reduced the atherogenicity of the lipid, lipoprotein, and apoprotein profile and insulin sensitivity [30, 31]. In animal studies, salsalate has been shown to reduce vascular injury in Zucker fatty rats and activate skeletal muscle thermogenesis and protect C57Bl/6J mice from a high-fat diet [32, 33]. In rodents, the effects of salsalate are thought to be due primarily to reducing inflammation; however, studies have shown that salsalate has metabolic effects beyond suppressing inflammation [34]. In diabetic mice, we have shown that salsalate alone partially improves peripheral neuropathy [5]. In this study, rats treated with salsalate alone or in combination with menhaden oil consumed about 40–50 mg salsalate per rat. Treatment of diabetic rats with salsalate alone tended to improve vascular reactivity of epineurial arterioles to acetylcholine and several neuropathy-related endpoints, but all these remained significantly impaired compared to control rats. Thus, it appears that salsalate alone was not as efficacious in diabetic rats compared to diabetic mice on improving peripheral neuropathy [5]. However, this study demonstrated for the first time that combining salsalate with menhaden oil provided greater efficacy toward improving vascular reactivity by epineurial arterioles to acetylcholine and calcitonin gene-related peptide.

Fish oils, such as menhaden oil, contain a high concentration of eicosapentaenoic and docosahexaenoic acids, and their consumption elevates the plasma concentration of proresolving inflammatory lipid mediators like resolvins E1 and D1, respectively [5]. This study extends our previous findings by demonstrating the effectiveness of different concentrations of menhaden oil with or without salsalate on resolvin D1 formation and alleviation of diabetic vascular and neural dysfunction in a type 2 diabetic rat model [5]. Data in Table 1 demonstrated that salsalate alone or in combination with menhaden oil reduces serum triglyceride levels. A similar outcome has been reported in humans with type 2 diabetes treated with salsalate [17]. Even though salsalate and 10% menhaden oil alone were minimally effective toward neuropathy-related endpoints, when combined, there was a significant improvement in motor nerve conduction velocity and thermal nociception (Table 2). Examining the effect of salsalate and menhaden oil on other neural endpoints impacted by diabetes, we found that treating diabetic rats with 25% menhaden oil alone significantly improved motor nerve conduction velocity and thermal nociception similar to the combination of salsalate and 10% menhaden oil, but sensory nerve conduction velocity, intraepidermal nerve fiber density, and corneal sensitivity remained significantly impaired. However, when salsalate was combined with 25% menhaden oil, all three of these neural endpoints were significantly improved (Table 2). Corneal nerve fiber length was significantly improved with 25% menhaden oil alone, and salsalate did not provide any additional benefit when combined with either 10% or 25% menhaden oil. Treating diabetic rats with 45% menhaden oil with or without salsalate provided maximal benefit toward these different neural endpoints.

Our study provides the first evaluation of the potential effects of salsalate alone or in combination with menhaden oil on diabetes-induced vascular dysfunction. Disrupted vascular function and reduced blood flow to peripheral nerves have long been considered a potential mechanism for diabetic peripheral neuropathy [35]. Our studies in both type 1 and type 2 diabetic rats have demonstrated that impaired vascular reactivity of epineurial arterioles, microvessels that provide blood flow to the sciatic nerve, precedes the development of nerve dysfunction, as identified by reduced nerve conduction velocity [26, 36]. Like in previous studies, vascular relaxation to acetylcholine or calcitonin gene-related peptide was decreased in diabetic rats. Treating diabetic rats with salsalate or 10% menhaden oil alone did not significantly improve vascular relaxation to acetylcholine. However, when diabetic rats were treated with salsalate and 10% menhaden oil, vascular relaxation to acetylcholine was significantly improved (Figure 2 and Table 3). A similar outcome was observed when vascular relaxation to calcitonin gene-related peptide was studied. Treating diabetic rats with salsalate or 10% menhaden oil alone did not improve vascular relaxation to calcitonin gene-related peptide (Figure 3 and Table 3). When diabetic rats were treated with the combination of salsalate and 10% menhaden, oil vascular relaxation to calcitonin gene-related peptide was moderately improved and the difference was not significantly different to control rats. Likewise, treating diabetic rats with 25% menhaden oil alone moderately improved vascular relaxation to calcitonin gene-related peptide but combining salsalate with 25% menhaden oil significantly improved vascular relaxation.

Some of the beneficial results of salsalate could be attributed to increased formation of resolvins when salsalate is combined with menhaden oil. Combining salsalate with 25% and 45% menhaden oil caused a significant increase in serum resolvin D1 levels (Table 1). Our studies demonstrated a significant improvement in some neural endpoints and vascular relaxation to acetylcholine when salsalate was combined with 10% menhaden oil. However, combining salsalate with 10% menhaden oil did not result in a significant increase in resolvin D1 levels in serum. This highlights some of the limitations of this study. We were limited to examining only resolvin D1 levels in serum in this study. We do not currently have the ability to measure resolvin E1 or neuroprotectin D1 in our serum samples. These determinations would require GC/MS, and we do not have access to this equipment, although a new core at the University of Iowa is being created that will provide this important capability in the near future. It is possible that resolvin E1 and/or neuroprotectin D1 are increased in diabetic rats treated with 10% menhaden oil and salsalate. To answer this question, it will be important to determine the combined levels of resolvins E1 and D1 as well as neuroprotectin D1 under the conditions used in this study. Previous studies have shown that treating diabetic mice with exogenous resolvin E1 or D1 improves peripheral neuropathy [5–7]. It has also been shown that resolvin D1 and neuroprotectin D1 can stimulate neurite outgrowth by dorsal root ganglion or trigeminal ganglion neurons in culture [6, 37]. These studies suggest that biological properties of resolvins and neuroprotectins toward inflammatory resolution and neural regeneration likely contribute to the beneficial effects of fish oil with or without salsalate on diabetes-induced vascular dysfunction and peripheral neuropathy. However, we cannot rule out other potential mechanisms that may also be contributing to the improved outcome of diabetic rats treated with menhaden oil and salsalate.

## 5. Conclusions

The principle finding of this study is that the beneficial dietary effects of fish oil on diabetic neurovascular dysfunction and peripheral neuropathy can be enhanced with the inclusion of salsalate in the diet. The beneficial effects of salsalate were most apparent at lower doses of dietary menhaden oil. Whether fish oil in combination with salsalate can provide benefit to patients suffering from diabetic peripheral neuropathy will require further investigation.

## Figures and Tables

**Table 1:** Effect of menhaden oil with or without salsalate on weight, chow consumed, serum triglycerides, free fatty acids, and cholesterol in type 2 diabetic Sprague Dawley rats.

Condition	Start weight (g)	End weight (g)	Chow consumed (g/kg body weight/day)	Blood glucose (mg/dl)	Serum triglycerides (mg/ml)	Serum-free fatty acids (mmol/L)	Serum cholesterol (mg/ml)
Control (12)	336 ± 3	494 ± 13	50.5 ± 3.0	147 ± 6	32.7 ± 5.8	0.13 ± 0.02	0.91 ± 0.02
Diabetic (12)	335 ± 2	439 ± 9	58.0 ± 3.8	478 ± 35^a^	192.3 ± 18.4^a^	0.37 ± 0.05^a^	1.95 ± 0.06^a^
Diabetic + salsalate (12)	333 ± 3	451 ± 12	48.0 ± 3.5	443 ± 40^a^	125.1 ± 25.9	0.36 ± 0.03^a^	1.73 ± 0.20^a^
Diabetic + menhaden oil 10% (12)	322 ± 3	420 ± 9	48.9 ± 3.3	469 ± 20^a^	246.4 ± 30.2^a^	0.42 ± 0.06^a^	1.53 ± 0.17^a^
Diabetic + menhaden oil 10% + salsalate (12)	326 ± 2	439 ± 21	50.9 ± 3.2	396 ± 43^a^	102.4 ± 25.0	0.32 ± 0.05^a^	1.55 ± 0.17^a^
Diabetic + menhaden oil 25% (12)	309 ± 4	426 ± 21	64.5 ± 4.6	500 ± 16^a^	316.9 ± 37.5^a^	0.41 ± 0.04^a^	2.81 ± 0.22^a^
Diabetic + menhaden oil 25% + salsalate (12)	305 ± 4	415 ± 14	58.2 ± 3.8	478 ± 39^a^	119.4 ± 26.2	0.35 ± 0.03^a^	1.78 ± 0.15^a^
Diabetic + menhaden oil 45% (12)	335 ± 3	459 ± 18	57.9 ± 4.3	426 ± 42^a^	82.6 ± 13.0^a,b^	0.40 ± 0.06^a^	1.96 ± 0.14^a^
Diabetic + menhaden oil 45% + salsalate (12)	315 ± 5	438 ± 14	67.2 ± 5.7	431 ± 35^a^	68.9 ± 18.6^b^	0.17 ± 0.04	1.33 ± 0.18

Data are presented as the mean ± SEM. ^a^*P* < 0.05 compared to control rats. ^b^*P* < 0.05 compared to diabetic rats. Parentheses indicate the number of experimental animals.

**Table 2:** Effect of menhaden oil with or without salsalate on motor and sensory nerve conduction velocity, thermal nociception, intraepidermal nerve fiber density, corneal sensitivity, and cornea nerve fiber length in type 2 diabetic Sprague Dawley rats.

Condition	Motor nerve conduction velocity (m/sec)	Sensory nerve conduction velocity (m/sec)	Thermal nociception (sec)	Intraepidermal nerve fiber density (profiles/mm)	Corneal sensitivity (cm)	Cornea nerve fiber length (mm/mm^2^)
Control (12)	55.7 ± 2.1	31.9 ± 0.5	11.9 ± 0.3	20.7 ± 0.7	5.77 ± 0.08	9.5 ± 0.5
Diabetic (12)	40.4 ± 1.4^a^	26.7 ± 0.6^a^	18.4 ± 0.5^a^	13.1 ± 0.8^a^	4.67 ± 0.22^a^	4.9 ± 0.4^a^
Diabetic + salsalate (12)	45.0 ± 1.9^a^	27.8 ± 0.4^a^	16.0 ± 1.0^a^	15.9 ± 0.4^a^	4.65 ± 0.20^a^	6.6 ± 0.5^a^
Diabetic + menhaden oil 10% (12)	44.1 ± 1.8^a^	27.3 ± 0.6^a^	15.2 ± 1.0	14.5 ± 0.9^a^	4.88 ± 0.19^a^	6.4 ± 0.5^a^
Diabetic + menhaden oil 10% + salsalate (12)	51.0 ± 2.0^b^	28.7 ± 0.6^a^	13.4 ± 1.2^b^	16.2 ± 0.06^a^	5.03 ± 0.22^a^	6.4 ± 0.5^a^
Diabetic + menhaden oil 25% (12)	47.2 ± 1.4^a^	28.3 ± 0.5^a^	12.3 ± 0.9^b^	15.6 ± 1.1^a^	4.81 ± 0.16^a^	8.1 ± 0.4^b^
Diabetic + menhaden oil 25% + salsalate (12)	49.2 ± 1.6^b^	29.7 ± 0.4^b^	11.2 ± 0.7^b^	18.9 ± 1.0^b^	5.58 ± 0.10^b^	8.5 ± 0.7^b^
Diabetic + menhaden oil 45% (12)	51.6 ± 1.4^b^	30.0 ± 0.4^b^	11.3 ± 0.4^b^	19.0 ± 1.2^b^	5.54 ± 0.13^b^	9.1 ± 0.6^b^
Diabetic + menhaden oil 45% + salsalate (12)	52.3 ± 1.2^b^	30.3 ± 0.5^b^	11.2 ± 0.7^b^	19.1 ± 0.6^b^	5.88 ± 0.06^b^	9.4 ± 0.6^b^

Data are presented as the mean ± SEM. ^a^*P* < 0.05 compared to control rats. ^b^*P* < 0.05 compared to diabetic rats. Parentheses indicate the number of experimental animals.

**Table 3:** Effect of menhaden oil with or without salsalate on log EC 50 for acetylcholine and calcitonin gene-related peptide-induced relaxation of epineurial arterioles of the sciatic nerve in type 2 diabetic Sprague Dawley rats.

Condition	Acetylcholine	Calcitonin gene-related peptide
Control (12)	−6.35 ± 0.11	−9.69 ± 0.04
Diabetic (12)	−4.04 ± 0.20^a^	−9.11 ± 0.08^a^
Diabetic + salsalate (12)	−5.12 ± 0.27^a^	−9.13 ± 0.07^a^
Diabetic + menhaden oil 10% (12)	−4.61 ± 0.44^a^	−9.11 ± 0.11^a^
Diabetic + menhaden oil 10% + salsalate (12)	−6.03 ± 0.36^b^	−9.38 ± 0.07
Diabetic + menhaden oil 25% (12)	−5.93 ± 0.34^b^	−9.23 ± 0.11
Diabetic + menhaden oil 25% + salsalate (12)	−6.14 ± 0.31^b^	−9.58 ± 0.10^b^
Diabetic + menhaden oil 45% (12)	−5.93 ± 0.26^b^	−9.46 ± 0.11^b^
Diabetic + menhaden oil 45% + salsalate (12)	−5.94 ± 0.25^b^	−9.56 ± 0.11^b^

Data are presented as the mean ± SEM. ^a^*P* < 0.05 compared to control rats. ^b^*P* < 0.05 compared to diabetic rats. Parentheses indicate the number of experimental animals.

## Data Availability

Upon written request from a responsible source, data pertaining to this study will be made available through the website for my laboratory. Work pertaining to this article was done in part as part of the official duties of the contact author to the federal government. Thus, data release will require a specific request.

## References

[1] Feldman E. L., Nave K. A., Jensen T. S., Bennett D. L. H. (2017). New horizons in diabetic neuropathy: mechanisms, bioenergetics, and pain. *Neuron*.

[2] Cortez M., Singleton J. R., Smith A. G. (2014). Glucose intolerance, metabolic syndrome, and neuropathy. *Handbook of Clinical Neurology*.

[3] Coppey L. J., Holmes A., Davidson E. P., Yorek M. A. (2012). Partial replacement with menhaden oil improves peripheral neuropathy in high-fat-fed low-dose streptozotocin type 2 diabetic rat. *Journal of Nutrition and Metabolism*.

[4] Coppey L. J., Davidson E. P., Obrosov A., Yorek M. A. (2015). Enriching the diet with menhaden oil improves peripheral neuropathy in streptozotocin-induced type 1 diabetic rats. *Journal of Neurophysiology*.

[5] Yorek M. S., Coppey L. J., Shevalye H., Obrosov A., Kardon R. H., Yorek M. A. (2016). Effect of treatment with salsalate, menhaden oil, combination of salsalate and menhaden oil, or resolvin D1 of C57Bl/6J type 1 diabetic mouse on neuropathic endpoints. *Journal of Nutrition and Metabolism*.

[6] Shevalye H., Yorek M. S., Coppey L. J. (2015). Effect of enriching the diet with menhaden oil or daily treatment with resolvin D1 on neuropathy in a mouse model of type 2 diabetes. *Journal of Neurophysiology*.

[7] Obrosov A., Coppey L. J., Shevalye H., Yorek M. A. (2017). Effect of fish oil vs. resolvin D1, E1, methyl esters of resolvins D1 or D2 on diabetic peripheral neuropathy. *Journal of Neurology & Neurophysiology*.

[8] Mori T. A. (2017). Marine OMEGA-3 fatty acids in the prevention of cardiovascular disease. *Fitoterapia*.

[9] Yanai H., Masui Y., Katsuyama H. (2018). An improvement of cardiovascular risk factors by omega-3 polyunsaturated fatty acids. *Journal of Clinical Medicine Research*.

[10] Zárate R., El Jaber-Vazdekis N., Tejera N., Pérez J. A., Rodríguez C. (2017). Significance of long chain polyunsaturated fatty acids in human health. *Clinical and Translational Medicine*.

[11] Yang L. G., Song Z. X., Yin H. (2016). Low n-6/n-3 PUFA ratio improves lipid metabolism, inflammation, oxidative stress and endothelial function in rats using plant oils as n-3 fatty acid source. *Lipids*.

[12] Serhan C. N., Petasis N. A. (2011). Resolvins and protectins in inflammation resolution. *Chemical Reviews*.

[13] Serhan C. N. (2014). Pro-resolving lipid mediators are leads for resolution physiology. *Nature*.

[14] Kromhout D., Giltay E. J., Geleijnse J. M., Alpha Omega Trial Group (2010). N–3 fatty acids and cardiovascular events after myocardial infarction. *New England Journal of Medicine*.

[15] Filipovic M. G., Aeschbacher S., Reiner M. F. (2018). Whole blood omega-3 fatty acid concentrations are inversely associated with blood pressure in young, healthy adults. *Journal of Hypertension*.

[16] Higgs G. A., Salmon J. A., Henderson B., Vane J. R. (1987). Pharmacokinetics of aspirin and salicylate in relation to inhibition of arachidonate cyclooxygenase and antiinflammatory activity. *Proceedings of the National Academy of Sciences of the United States of America*.

[17] Goldfine A. B., Fonseca V., Jablonski K. A. (2013). Salicylate (Salsalate) in patients with type 2 diabetes: a randomized trial. *Annals of Internal Medicine*.

[18] Faghihimani E., Aminorroaya A., Rezvanian H., Adibi P., Ismail-Beigi F., Amini M. (2013). Salsalate improves glycemic control in patients with newly diagnosed type 2 diabetes. *Acta Diabetologica*.

[19] Cryer B., Goldschmiedt M., Redfern J. S., Feldman M. (1990). Comparison of salsalate and aspirin on mucosal injury and gastroduodenal mucosal prostaglandins. *Gastroenterology*.

[20] Davidson E. P., Coppey L. J., Holmes A., Yorek M. A. (2012). Effect of inhibition of angiotensin converting enzyme and/or neutral endopeptidase on vascular and neural complications in high fat fed/low dose streptozotocin-diabetic rats. *European Journal of Pharmacology*.

[21] Davidson E. P., Coppey L. J., Holmes A., Yorek M. A. (2012). Changes in corneal innervation and sensitivity and acetylcholine-mediated vascular relaxation of the posterior ciliary artery in a type 2 diabetic rat. *Investigative Ophthalmology & Visual Science*.

[22] Pritchard N., Edwards K., Vagenas D., Russell A. W., Malik R. A., Efron N. (2012). Corneal sensitivity is related to established measures of diabetic peripheral neuropathy. *Clinical and Experimental Optometry*.

[23] Malik R. A., Kallinikos P., Abbott C. A. (2003). Corneal confocal microscopy: a non-invasive surrogate of nerve fibre damage and repair in diabetic patients. *Diabetologia*.

[24] Davidson E. P., Coppey L. J., Kardon R. H., Yorek M. A. (2014). Differences and similarities in development of corneal nerve damage and peripheral neuropathy and in diet-induced obesity and type 2 diabetic rats. *Investigative Ophthalmology & Visual Science*.

[25] Yorek M. S., Davidson E. P., Poolman P. (2016). Corneal sensitivity to hyperosmolar eye drops: a novel behavioral assay to assess diabetic peripheral neuropathy. *Investigative Ophthalmology & Visual Science*.

[26] Coppey L. J., Davidson E. P., Dunlap J. A., Lund D. D., Yorek M. A. (2000). Slowing of motor nerve conduction velocity in streptozotocin-induced diabetic rats is preceded by impaired vasodilation in arterioles that overlie the sciatic nerve. *International Journal of Experimental Diabetes Research*.

[27] Yorek M. A., Coppey L. J., Gellett J. S., Davidson E. P. (2004). Sensory nerve innervation of epineurial arterioles of the sciatic nerve containing calcitonin gene-related peptide: effect of streptozotocin-induced diabetes. *Experimental Diabesity Research*.

[28] Reed M. J., Meszaros K., Entes L. J. (2000). A new rat model of type 2 diabetes: the fat-fed, streptozotocin-treated rat. *Metabolism*.

[29] Srinivasan K., Viswanad B., Asrat L., Kaul C. L., Ramarao P. (2005). Combination of high-fat diet-fed and low-dose streptozotocin-treated rat: a model for type 2 diabetes and pharmacological screening. *Pharmacological Research*.

[30] Alderete T. L., Sattler F. R., Richey J. M. (2015). Salsalate treatment improves glycemia without altering adipose tissue in nondiabetic obese Hispanics. *Obesity*.

[31] Ariel D., Kim S. H., Liu A. (2015). Salsalate-induced changes in lipid, lipoprotein, and apoprotein concentrations in overweight or obese, insulin-resistant, nondiabetic individuals. *Journal of Clinical Lipidology*.

[32] Murthy S. N., Desouza C. V., Bost N. W. (2010). Effects of salsalate therapy on recovery from vascular injury in female Zucker fatty rats. *Diabetes*.

[33] Nie L., Yuan X. L., Jiang K. T. (2017). Salsalate activates skeletal muscle thermogenesis and protects mice from high-fat diet induced metabolic dysfunction. *eBioMedicine*.

[34] Trnovska J., Silhavy J., Kuda O. (2017). Salsalate ameliorates metabolic disturbances by reducing inflammation in spontaneously hypertensive rats expressing human C-reactive protein and by activating brown adipose tissue in nontransgenic controls. *PLoS One*.

[35] Yorek M. A. (2015). Vascular impairment of epineurial arterioles of the sciatic nerve: implications for diabetic peripheral neuropathy. *The Review of Diabetic Studies*.

[36] Coppey L. J., Gellett J. S., Davidson E. P., Dunlap J. A., Yorek M. A. (2002). Changes in endoneurial blood flow, motor nerve conduction velocity and vascular relaxation of epineurial arterioles of the sciatic nerve in ZDF-obese diabetic rats. *Diabetes/Metabolism Research and Reviews*.

[37] Cortina M. S., He J., Russ T., Bazan N. G., Bazan H. E. P. (2013). Neuroprotectin D1 restores corneal nerve integrity and function after damage from experimental surgery. *Investigative Ophthalmology & Visual Science*.

